# Truffle renaissance in Poland – history, present and prospects

**DOI:** 10.1186/s13002-017-0163-x

**Published:** 2017-06-15

**Authors:** Aleksandra Rosa-Gruszecka, Dorota Hilszczańska, Wojciech Gil, Bogusław Kosel

**Affiliations:** 1 Department of Forest Protection, Forest Research Institute, Sękocin Stary, Braci Leśnej 3, 05-090 Raszyn, Poland; 2 Department of Forest Ecology, Forest Research Institute, Sękocin Stary, Braci Leśnej 3, 05-090 Raszyn, Poland; 3 Department of Silviculture and Genetics of Forest Trees, Forest Research Institute, Sękocin Stary, Braci Leśnej 3, 05-090 Raszyn, Poland; 4 Faculty of History and Sociology, Bialystok University, Plac Uniwersytecki 1, 15-420 Białystok, Poland

**Keywords:** Ethnomycology, Historical data, Truffles, Cuisine, Poland

## Abstract

The use of truffles in Poland has a long tradition, yet due to some historical aspects, this knowledge was lost. Currently, truffles and truffle orchards are again receiving attention, and thanks to, e.g., historical data, they have solid foundations to be established. Publications relating to truffles between 1661 and 2017 were searched for in international and national databases, such as the database of PhD theses, Google Scholar, and catalogues of the National Library of Poland, the Jagiellonian Digital Library, the University Library of J. Giedroyc in Bialystok and the Lower Silesian Digital Library (DBC). A very meticulous survey of the literature on truffles showed that truffles have been known since at least 1661. In the 18th century, the fungi were considered a non-timber forest product. It is interesting to mention the impact of Polish Count Michał Jan Borch in understanding the nature of truffles. The whitish truffle (*Tuber borchii*) is named after him. The greatest number of publications regarding truffles can be observed at the first half of the 19th and 20th centuries. The fungi were present not only in cookbooks but also in scientific literature, and aspects of their ecology and medicinal use are considered. The “dark ages” for truffles, mainly for social reasons, occurred after the Second World War. In tough times, when Poland was under Soviet communist control (1945–1989), truffles as a luxurious product have been completely forgotten. However, at the end of the 20th century, truffles started receiving attention in Polish society. Yet, the real awakening began in the first decade of the twenty-first century when the first truffle orchards were established. One of them has already produced the first fruit bodies of summer truffle (*Tuber aestivum*). Truffles have been present in Polish culture for centuries. Their renaissance indicates the need for fostering sustainable agroforestry-centred initiatives aimed at helping truffle growers in growing the precious fungi and thus meeting market demands.

## Background

Truffles (*Tuber* spp.) are hypogeous fungi belonging to the Pezizales (Ascomycota), a large group of symbiotic fungi growing with the roots (ectomycorrhiza) of several vascular plant species (angiosperms and gymnosperms). Some species of truffles, such as *Tuber magnatum* Pico (white truffle) and *Tuber melanosporum* Vittad. (black truffle), are the most valued and expensive due to their taste and aroma [1]. On average, the worldwide prices of *T. magnatum* range from €1200 to €4000 per kg depending on harvest [2]. The high prices are due to distinctive features of the special truffle and its insufficient provision [3]. This species is hard to grow on man-made plantations, so it is harvested mainly in natural stands [4]. To date, *T. melanosporum*, *T. brumale* and *T. aestivum* have been growing on plantations, yet in the case of *T. magnatum*, there is still a lack of cultivation methodology [5]. The natural distribution of *T. magnatum* is limited to some locations in Italy [2], Hungary [6], Slovenia [7] and Croatia [8].

The other appreciated species of truffles, *T. melanosporum,* is being cultivated worldwide [9]. Truffle orchards have been established in the southern hemisphere, and truffles are present in the market year-round [10]. Demand for black truffle (*T. melanosporum*) has stimulated research on the species. Hence, the biology of the species at the genomic level [11] is better understood than that of other truffle species. Soil and climate conditions conducive to *T. melanosporum* development are well known [12, 13]. However, in the last decade, a decreased yield of this species has been observed in Europe. Some authors combine this fact with climate warming (for instance, [14–16]). The increasing attention towards the highly appreciated and commercialized hypogeous fungi has led to intensification of the research on truffles to better understand their life cycle [1].

The first evidence for the culinary use of truffles by people inhabiting the eastern coast of the Mediterranean Sea comes from the Bronze era [17]. Hypogeous Ascomycetes of the genus *Tirmania* Chatin and *Terfezia* Tul. & C. Tul., known as desert truffles, have nourished the tribes of the Sahara [18]. Hypogeous fungi were known and eaten by ancient Babylonians, Etruscans, Egyptians, Greeks and Romans [19, 20]. In ancient times, truffles were a great mystery to scientists and common people because it was unknown where truffles came from. For example, according to the Greek biographer Plutarch (46–120 A.D.), a truffle was a conglomeration produced by the action of lightning, warmth, and water on the soil. Dioscorides (40–90 A. D.), the Greek physician and pharmacologist, thought that the truffle was a tuberous root. Theophrateous, the Greek philosopher (c. 370–280 B. C.), described truffles as plants without root, stem, branch, bud, leaf, flower, or fruit and with neither bark, pith, fibres, nor veins [21].

It is thought that the popularity of truffles during the Middle Ages was far less than that in ancient times [22]. However, from the latter era come precious works that describe methods of searching for truffles, referred to by some authors as ‘hunting for truffles’ (fr. *chasse aux truffes*, wł. *caccia al tartufo*, ang. *truffle hunting*) [22, 23]. Hunting for truffles in Italy using pigs and dogs has been depicted by the papal historian Bartolomeo Platina and the painter Ambrogio Lorenzetti [19].

At the beginning of the 18th century, truffles regained popularity and were included on menus by the French and Italians [22]. During the first decade of the 18th century, French botanist and physician Joseph Pierre de Tournefort and the pharmacist and botanist Claude-Joseph Geoffroy made observations that helped us recognize the nature of truffles. Geoffrey helped settle the botanical confusion surrounding the truffle, and in a 1711 paper titled “Vegetation de la Truffe”, he classified it among fungi [21]. Geoffroy’s observations were confirmed by Pier Antonio Micheli, the Italian botanist who provided the description of “seeds” (spores) in truffles. In his publication, *Nuova plantarum genera* [24], he noted that the spores developed inside sacks (asci). Over a hundred years later, Carlo Vittadini [25] and the Tulasne brothers [26] firmly established the scientific study of truffles. The latter researchers are considered the founders of modern mycology [21, 27].

Cultivation of truffles began at the turn of 18th and 19th centuries in France and Italy [28]. The first commonly used method of truffle orchard establishment is known as Talon’s technique. In 1808, Talon proposed the idea of transplanting some seedlings that he had collected at the foot of oak trees known to host truffles in their root system. His technique was the mainstay of the black truffle industry for more than 150 years, although he was not aware that the success of truffle fructification depended on the mycorrhiza. The phenomenon of mycorrhiza has been described by Franciszek Kamieński [29] and Albert Benjamin Frank [30] introduced the term *Mykorrhizen* (from the Greek *myko* - fungus, *rhiza* - root) in 1885. Talon’s method of truffle orchard establishment was in use until the early 1970s, when French and Italian scientists developed the technique of nursery seedling inoculation. In 1973, the first seedlings inoculated with *T. melanosporum* appeared on the market [31].

Despite the technological progress since the 1980s, a decrease in productivity of the plantation is being observed [20]. Some researchers attribute this decrease to changes in rural land forest use [20] and others attribute it to changes in climate [32]. The decreasing supply and rising prices of truffles have provided an enormous incentive for research on truffle cultivation. At present, truffle orchards are being established all over the world, including in non-traditional areas and countries, for example, the United States, New Zealand, and Australia [13, 33].

In the mycological databases, such as Mycobank and Index Fungorum Mycobank, taxonomic details of more than 200 truffle species from all over the world are given, but only 70 species are fully verified [34]. On the other hand, Bonito and co-authors [35] reported at least 180 species of truffles. According to Ceruti [36], 28 species of *Tuber* are present in Europe. In 2012, another species of the genus was added, *Tuber cistophilum*, and its identity was confirmed using molecular tools [37].

In Poland, the presence of some species that are in great demand by the food market, such as *Tuber macrosporum* Vittad., *T. mesentericum* Vittad., *T. borchii* Vittad. and *T. aestivum* var. *uncinatum* Chatin, has been confirmed [38–40]. However, due to a “dark age” in Polish history (Soviet communist regime) knowledge about truffles and their occurrence has been lost for over half a century. Thankfully, research on these fungi and their use is currently undergoing a renaissance. Therefore, basing on historical data, the aim of this work is to:

- show that truffles has been present in Polish culture for ages,
- indicate the great potential of Poland in truffle collection and their culinary usage, as well as possibilities to grow the ultimate fungi.

## Methods

To access to the maximum amount of data on truffle use in the past, detailed analysis was conducted using articles and books. International and national databases, such as the database of PhD theses, Google Scholar, and catalogues of the National Library of Poland, the Jagiellonian Digital Library, the University Library of J. Giedroyc in Bialystok and the Lower Silesian Digital Library (DBC) were checked. The overall search pattern covered not only the title, abstract and keywords but also the content concerning truffles or *Tuber*. No restrictions regarding the language of the publications consulted were imposed.

Each cited publication is indicated by letters (Table 1) depicting the content of a given publication: C – culinary and medicine use, methods of fruit bodies conservation; E – economical aspects, financial benefits from truffle hunting; L – belles-letters (poems, tales, songs, etc.); H – truffle hunting methods, dogs training; O – ecology, environmental conditions conducive to truffle occurrence; S – diversity of truffle species, truffle protection; T– truffle orchard establishment; X – others. Table 2 lists cookbooks and indicates the number of recipes with truffles.

**Table 1:** Truffles in Polish literature

	No.	Date of publication	Author(s)	Ref.	Concerns^a^							
					O	C	S	H	E	T	L	X
The 2nd half of the 17th century	1.	1661	Pasek	[108]		+						
	2.	1682	Czerniecki	[41]		+						
						2						
The 1st half of the 18th century	1.	1719	Hubert	[42]				+	+			
								1	1			
The 2nd half of the 18th century	1.	1780	Borch	[79]	+			+	+	+		
	2.	1778	Kluk	[43]	+	+			+			
	3.	1783	Wielądko	[50]		+						
	4.	1786	Kluk	[44]		+	+					
	5.	1799	Jundziłł	[69]	+	+	+	+	+	+		
					3	4	2	2	3	2		
The 1st half of the 19th century	1.	1822	Szczepański	[51]		+						
	2.	1823	Bobiatyński	[45]	+			+				
	3.	1828	Bornholz	[80]	+	+	+	+	+	+		
	4.	1829	Bornholz	[81]	+	+	+	+	+	+		
	5.	1830	Bornholz	[82]	+	+	+	+	+	+		
	6.	1830	Gołębiowski	[52]		+						
	7.	1831	Gołębiowski	[46]				+				
	8.	1835	Zawadzki	[70]	+							
	9.	1838	Dąbkiewicz	[53]		+						
	10.	1838	Leśniewski	[54]		+						
	11.	1839	Szytler	[23]	+	+	+	+	+			
	12.	1840	Kitowicz	[109]		+						
	13.	1840	Kraszewski	[56]		+						
	14.	1841	Pisulewski	[71]	+							
	15.	1845	Gerald-Wyżycki	[72]	+	+	+	+				
	16.	1847	Plater	[74]	+	+		+				
	17.	1849	Czerwiakowski	[73]	+	+	+	+				
					10	13	6	9	4	3		
The 2nd half of the 19th century	1.	1853	Wydrzyński	[110]	+			+	+			
	2.	1856	Leśniewska	[55]		+						
	3.	1859	Belke	[75]	+							
	4.	1859	Lelewel	[76]		+						
	5.	1860	Bill	[77]	+		+					
	6.	1865	Kurowski	[111]	+			+				
	7.	1867	Berdau	[57]	+	+	+	+		+		
	8.	1871	Ćwierczakiewicz	[103]		+						
	9.	1888	Błoński	[47]	+		+	+	+	+		
	10.	1889	Biełozierska	[58]		+						
	11.	1892	Rewiński	[48]				+				
	12.	1894	Aleksandrowicz, Błoński	[112]	+		+					
	13.	1895	Gawarecki	[83]						+		
	14.	1897	Spausta	[78]	+		+	+	+	+		
					8	4	5	6	3	4		
The 1st half of the 20th century	1.	1900	Schnaider	[64]		+						
	2.	1901	Doleżan	[113]	+	+	+	+	+			
	3.	1903	Norkowska	[59]		+						
	4.	1905	Chełkowski	[114]	+	+	+		+			
	5.	1905	Niewiarowska	[60]		+						
	6.	1910	Hildt	[115]	+							
	7.	1910	Ochorowicz-Monatowa	[116]		+						
	8.	1911	Kurcyusz	[87]	+							
	9.	1914	Owoczyńska	[61]		+						
	10.	1917	Teodorowicz	[86]	+	+				+		
	11.	1923	Blei	[66]							+	
	12.	1928	Swoboda	[62]		+						
	13.	1932	Kobylański	[117]	+	+	+	+		+		
	14.	1932	Śleżańska	[63]		+						
	15.	1933	Szulczewski	[85]		+			+			
	16.	1938	Orłoś	[118]	+	+		+	+			
	17.	1947	Orłoś	[90]	+		+	+				
	18.	1948	Dąbrowska	[119]		+					+	
					8	15	4	4	4	2	2	
The 2nd half of the 20th century	1.	1950	Biegańska-Hornowska	[91]		+		+	+			
	2.	1953	Lubelska	[92]	+		+					
	3.	1953	Orłoś	[120]			+					
	4.	1958	Brzechwa	[67]							+	
	5.	1970	Lemnis, Vitry	[121]		+						
	6.	1970	Ihnatowicz	[122]		+						
	7.	1975	Kuchowicz	[49]		+						
	8.	1986	Ścisłowski	[68]							+	
	9.	1988	Grzywacz	[123]			+					
	10.	1988	Ławrynowicz	[93]	+		+	+				
	11.	1991	Kowecka	[124]				+				
	12.	1999	Ławrynowicz	[38]	+		+					
					3	4	5	3	1		2	
The 1st half of the 21st century	1.	2008	Hilszczańska et al.	[97]	+		+					
	2.	2008	Ławrynowicz et al.	[94]	+		+					
	3.	2009	Hilszczańska	[99]	+		+		+			
	4.	2009	Ławrynowicz	[95]	+		+					
	5.	2013	Gajos, Hilszczańska	[98]	+	+						+
	6.	2013	Hilszczańska et al.	[40]	+		+					
	7.	2014	Gajos et al.	[65]		+						
	8.	2014	Hilszczańska et al.	[100]	+		+					
	9.	2014	Łuczaj, Köhler	[84]			+					+
	10.	2014	Rosa-Gruszecka et al.	[107]	+		+					
	11.	2015	Hilszczańska	[101]	+		+			+		
	12.	2015	Jankiewicz et al.	[125]	+							
	13.	2016	Byk et al.	[88]	+							
	14.	2016	Hilszczańska et al.	[102]	+		+			+		
	15.	2016	Hilszczańska	[126]	+	+	+	+	+	+		
	16.	2017	Rosa-Gruszecka et al.	[89]	+				+			
					14	3	11	1	3	3		2
Total:	85 publications				46	45	33	26	19	14	4	2

^a^C – culinary, medicine, methods of fruit bodies conservation; E – economical aspects, financial benefits from truffle hunting; L – belles-letters (poems, tales, songs, etc.); H – truffle hunting methods, dogs training; O – ecology, environmental conditions conducive for truffle occurrence; S – diversity of truffle species, truffle protection; T– truffle orchard establishing; X – others

**Table 2:** Truffles in Polish cookbooks

No.	Century	Date of publication	Authors	Reference	Number of recipes with truffles
1.	17th	1682	Czerniecki	[41]	2
2.	18th	1783	Wielądko	[50]	14
3.	19th	1822	Szczepański	[51]	3
4.		1838	Dąbkiewicz	[53]	1
5.		1838	Leśniewski	[54]	3
6.		1856	Leśniewska	[55]	9
7.		1871	Ćwierczakiewicz	[103]	7
8.		1889	Biełozierska	[58]	1
9.	20th (before the Second World War)	1903	Norkowska	[59]	41
10.		1905	Niewiarowska	[60]	2
11.		1910	Ochorowicz-Monatowa	[116]	18
12.		1914	Owoczyńska	[61]	14
13.		1928	Swoboda	[62]	4
14.		1932	Śleżańska	[63]	3
15.	20th (after the Second World War)	1968	Berger et al.	[127]	0
16.		1983	Zawistowska, Krzyżanowska	[128]	0
17.		1987	Czerni	[129]	0
18.		1989	Bytnerowiczowa	[130]	0
19.		1991	Górska	[131]	0
20.	21st	2013	Brodnicki, Okrasa	[132]	0

## Results

### From the 17th century to the second world war

The first recipes for dishes with truffles can be found in the first Polish cookbook written by Stanislaw Czerniecki [41], who worked as a chamberlain at Michał Lubomirski’s court in Kraków. The book contains recipes for fish stuffed with truffles and truffles baked in the ashes. At that time, nobles in Poland used to hunt for truffles with dogs [42], which indicates the prevalence of these fungi. Kluk indicated the truffle in his publications [43, 44] as one of many forest products. Following publications from the 19th century, the data on truffle hunting with trained dogs can be found [23, 45–48].

At the time of King Augustus II the Strong (1697–1706, 1709–1733) and King Augustus III (1733–1763), truffles were used as a condiment to Polish dishes, and new ones came to Poland with French chefs [41]. Kuchowicz [49], describing Polish customs in the 17th and 18th centuries, also noted that dishes with truffle were served. In another Polish cookbook written by Wielądko [50], a recipe for chicken with truffles can be found. In the 19th century and at the beginning of the 20th century, the comments on truffles as well as the number of recipes with truffles indicate the culinary use of truffle [51–63].

Kluk, in his work [43], wrote about truffles as a product of lower forest utility: ‘here, I could assign the truffles, especially those growing in the oak forests, as valuable and prized at nobles’ tables if I would not consider how hard it is to find them and that they are not typical of all types of forest’. Later, Kluk [44] reported about the medicinal properties of truffles and its potions’ components. Over a century later, Schnaider [64] depicted the use of truffles by the Huculs (an ethnic group living in the southern part of Ukraine and in the north of Romania), who used truffles to mitigate heart problems. Contemporary data confirmed the medicinal properties of truffles in this context [65].

A few writers and poets have written about truffles. Usually, a truffle appeared as a metaphor for love in the Manual of Love Franz Blei [66] or as poems and epigrams for children, for example, those of Brzechwa [67] and Ścisłowski [68].

In the 18th and 19th centuries, some authors described domestic truffles and places of their growth [53, 69–78]. It was also during this time when Polish Count Michał Jan Borch [79], scientist and naturalist, started searching for methods of truffle cultivation. The whitish truffle (*T. borchii*) is named after him.

The first study on the establishment of truffle orchards, which occurred in Poland, comes from a translation from German from the book by Bornholz [80]. Another publication by the author was published in Polish journals, such as *Piast czyli pamiętnik technologiczny* [81] and in *Sylwan* [82], and it shows the interest in cultivation of truffles at that time. Some hints on hunting for truffles and growing truffles were also published by Gawarecki [83]. Due to a survey done in 1883 on plants’ common names incited by Rostafiński (the professor of botany of Jagiellonian University in Krakow) [84], the presence of hypogeous fungi, very likely *Tuber* spp. or *Choiromyces meandriformis* in southern and eastern parts of Poland, was confirmed. Interest in truffles is given also by Szulczewski [85] who reported selling three species of fungi, viz.: *Scleroderma vulgare*, *S. verrucosum* and *Rhizopogon luteolus* as true truffles (black or white, respectively).

Rich notes dedicated to truffle occurrence, cultivation and culinary use are also given by Teodorowicz [86]. The author emphasized the phenomenon of symbiosis linking truffles and trees. Mutualistic characters of plants and animals’ relation, including flies and beetles, which are vectors of truffles are highlighted by Kurcyusz [87]. This theme was rather rarely present in Polish research and came back over 100 years later [88, 89].

### The aftermath of the second world war

After the Second World War truffles were forgotten due to some changes of social and cultural character as well as changes of forest management. Primary factors determining truffle forgetfulness are given in further part of this article.

In the late 40s of the last century, despite the rich body of historical records on truffle, their presence in Poland was questioned [90]. However, at the beginning of the 1950s, some authors confirmed their occurrence [91, 92]. Lubelska [92] noted the sites of seven truffles species, including *T. aestivum, T. borchii, T. rufum, T. puberulum, T. rapeodorum* and *T. melanospermum*.

At the beginning of the 1980s, truffles began receiving attention again. Ławrynowicz [93] provided a scientific basis and summarized the knowledge about underground fungi. She described nineteen genera of ascomycetes that produce subterranean fruiting bodies, including *Tuber* spp. Data on the differentiation and classification of 21 European truffle species are given in this book. Based on available herbarium material, Ławrynowicz confirmed eight truffle species in Poland, among which only two are considered to be culinary prized, viz. *T. mesentericum and T. borchii.*

In 1981, *T. mesentericum* was found by Ławrynowicz for the first time at one location [38]. Currently, there are five known locations of this species in southern Poland [94, 95]. In Poland, this species is the only one among the other truffles which is under species’ legal protection [96].

In 2007, research on truffles conducted by Italian scientists revealed the presence of *T. aestivum* and other truffle species in Nida Basin [97]. The identity of *T. aestivum, T. maculatum,* and *T. fulgens* was genetically confirmed, and the sequences are deposited in NCBI (for example, [39]). Another economically and culinarily valid species of truffle*, T. macrosporum,* was found in the autumn of 2012 [40]. Its sequences were also deposited in the above-mentioned Gene Bank.

Ongoing research on truffles considers different aspects of the truffle’s life, and the number of publication dealing with the subject has been increasing [98]. In the last decade, the first Polish truffle orchards with seedlings inoculated from a native inoculum have been established, and in the oldest one, which was established in 2008, fruit bodies of *T. aestivum* occurred last autumn. The dates of the research done in the last decade were published by Hilszczańska and co-workers [97–102].

### Truffles in Polish literature through the ages

**Fig. 1:**
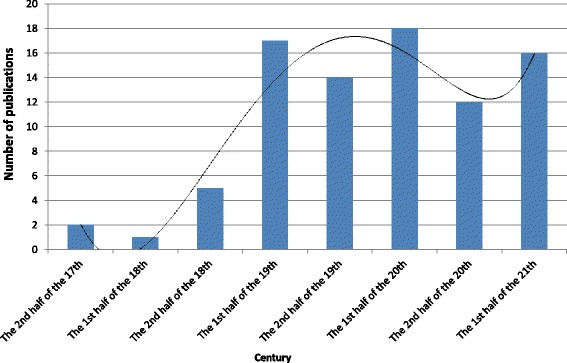
Number of publications on truffles in Poland from the 17th century to today

The knowledge of truffles dates back to the 17th and 18th centuries. At the beginning of the 19th century, the number of publications about truffles increased, which reflected the interest of Polish society in the fungi. During the 19th century, 31 articles were published on the topic of truffles. Similarly, in the 20th century, 30 works concerning truffles have been published, with most of them published in the first half of the 20th century (Fig. 1). Table 1 shows the list of publications dealing with truffles published between 1661 and 2017.

The main topics of these articles are the ecology of truffles and the environmental conditions conducive to their development, as well as their distribution. A great number of publications contain culinary and medicinal properties of the fungi, as well as methods for their storage. Topics on the culinary truffles appear not only in cookbooks but also in publications about the customs of the court over the centuries. In the 19th century, the focus was often on hunting for truffles and placing dogs for exploration, but today, most attention is paid to both the ecology and the protection aspects.

**Fig. 2:**
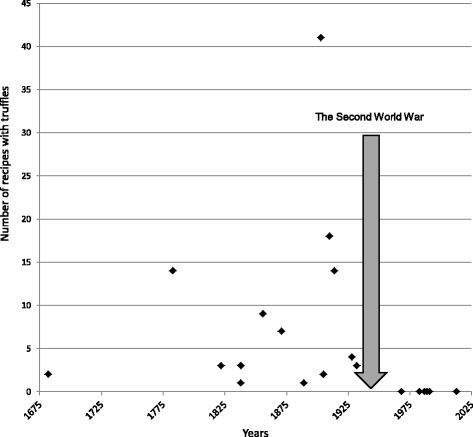
Number of recipes with truffles in Polish cookbooks from the 17th century until today

It has been observed that the number of cookbooks with recipes for dishes with truffles, as well as the number of stand-alone recipes, has increased over the centuries (Fig. 2). Some of them were subsequently reproduced, for example, the recipe for truffle sauce [54, 55, 60] or the recipe for the sirloin with truffle sauce [59, 60, 103]. Some names of the dishes indicate the preferences of Polish aristocrats, for instance, ‘Perch à la Radziwiłł’, or ‘Chop à la Radziwiłł’ [59]. The golden age of truffles in Poland ended with the Second World War. Their use over the centuries is shown in Table 2.

## Conclusions and future perspectives

Tracing the historical data on truffles indicates that truffles have been present in Polish culture for centuries. The fungi were valued and initially served at the royal court and aristocrats’ tables, such as those of Radziwiłłs, Branickis, and Lubomirskis. Gradually, the culinary use of truffles went into broader society levels, and hunting for truffles, together with traditional hunting, became popular. To train dogs for truffle finding, professionals were employed. Disappearance of truffles as a well-known delicacy after the Second World War was due to some factors of a socio-economic nature:

1. Changes in forest cover. After the Second World War, forests comprised only 20.8% of Polish territory. Unfavourable conditions for fruiting truffles included changes in species composition, age structure of stands and changes of forest management. For example, undergrowth shading the forest floor was more common due to the cessation of grazing in forests.
2. Changes in the structure of forest ownership and use. The disappearance of traditional types of forest use, such as cattle grazing and collection of brushwood.
3. Changes at the society level due to war and the great loss of Polish citizens, especially the loss of Polish aristocracy and intelligentsia, including foresters, or social groups with the most knowledge and practice regarding collection, use and cultivation of truffles; emigration and migration of population from rural to urban areas.
4. The communist regime promoted “pork chop and carp” as the food for the ‘working class’ rather than the traditional delicacies of Polish cuisine. Truffles as a luxury product for the nobility were not welcomed by new authorities.

In Poland, research on the factors determining truffles’ cultivation is still in the pioneering stage [100]. Truffles in Poland are considered as rare fungi and many species remain undiscovered. Some of them, *Tuber aestivum*, for example, have the status of extinct and missing species on the Polish Red List of Plants and Fungi [104]. The area of soils which are conducive to truffle’ development is rather small. According to Krasowicz et al. [105] rendzinas and pararendzinas are only 1.1% of all type of soils. Moreover, a great part of the soil is used for agricultural purposes, and only a small portion is covered with forests. For instance, around the Forest District in Pińczów (Nida Basin), the location where research on truffles has been occurring since 2007 [97, 100], forest cover is only 10%. The share of stands on rendzic soil is only 7% of the forest area [106] (Plan Urządzenia Gospodarstwa Leśnego Nadleśnictwa Pińczów na lata 2013─2022).

Although it seems that scarcity of soils conducive to truffle growth could be a serious obstacle to promote and establish truffle orchards in Poland, the results of our pioneering work brings hope. We have obtained fruit bodies of *T. aestivum* after 8 years in truffle orchards established in eastern Poland [102]. Two other orchards are cared for by the Forest Research Institute, and new orchards are established every year by individual entrepreneurs. Currently, the priority in our work is to bring the truffles back to Polish society and to achieve in situ protection of *Tuber* fungi [97, 100, 107].
